# Selective autophagy: adding precision in plant immunity

**DOI:** 10.1042/EBC20210063

**Published:** 2022-08-05

**Authors:** Jia Xuan Leong, Gautier Langin, Suayib Üstün

**Affiliations:** 1 University of Tübingen, Center for Plant Molecular Biology (ZMBP), 72076 Tübingen, Germany; 2 Faculty of Biology & Biotechnology, Ruhr-University Bochum, 44780 Bochum, Germany

**Keywords:** Autophagy, Effectors, Immunity

## Abstract

Plant immunity is antagonized by pathogenic effectors during interactions with bacteria, viruses or oomycetes. These effectors target core plant processes to promote infection. One such core plant process is autophagy, a conserved proteolytic pathway involved in ensuring cellular homeostasis. It involves the formation of autophagosomes around proteins destined for autophagic degradation. Many cellular components from organelles, aggregates, inactive or misfolded proteins have been found to be degraded via autophagy. Increasing evidence points to a high degree of specificity during the targeting of these components, strengthening the idea of selective autophagy. Selective autophagy receptors bridge the gap between target proteins and the forming autophagosome. To achieve this, the receptors are able to recognize specifically their target proteins in a ubiquitin-dependent or -independent manner, and to bind to ATG8 via canonical or non-canonical ATG8-interacting motifs. Some receptors have also been shown to require oligomerization to achieve their function in autophagic degradation. We summarize the recent advances in the role of selective autophagy in plant immunity and highlight NBR1 as a key player. However, not many selective autophagy receptors, especially those functioning in immunity, have been characterized in plants. We propose an *in silico* approach to identify novel receptors, by screening the *Arabidopsis* proteome for proteins containing features theoretically needed for a selective autophagy receptor. To corroborate these data, the transcript levels of these proteins during immune response are also investigated using public databases. We further highlight the novel perspectives and applications introduced by immunity-related selective autophagy studies, demonstrating its importance in research.

## Introduction

Plant immunity centers around the plant-pathogen interface, where an intriguing arms race occurs at the molecular level. Plants defend themselves against pathogens using their innate immunity, where broad resistance to pathogens is conferred by pathogen-associated-molecular-pattern (PAMP)-triggered immunity (PTI), which involves recognition of conserved molecules shared by pathogens by surface-localized pattern-recognition receptors (PRRs) [1–3]. For a successful infection, plant pathogens such as bacteria, fungi or oomycetes colonize the plant extracellular space and secrete effector proteins into the host cell cytoplasm, which then target essential pathways in the plant [2,4,5]. Plant viruses are rather obligate intracellular parasites, but likewise utilize effectors for manipulating host processes from within the plant cell, and to facilitate cell-to-cell movement [6–8]. Effectors have been shown to target key plant processes such as PTI signaling, cytoskeleton localization and endocytic trafficking, in a process termed “effector-targeted pathways (ETP)” [4]. Effectors can in turn be recognized by nucleotide-binding leucine-rich repeat receptors (NLRs), which guard host immune components and induce effector-triggered immunity (ETI) [1–3].

As cellular processes are mediated to a great extent by proteins, plant proteostasis i.e. the homeostasis of the plant proteome is an important factor to consider in immunity. Proteostasis is the dynamic and constant interplay between protein synthesis and degradation, which can be influenced by factors such as protein translation, folding, localization, post-translational modifications, proteasome and autophagic activity [9]. A major hub where effectors converge for ETP are the main protein degradation pathways, autophagy and the ubiquitin–proteasome system (UPS) [5]. Both proteolytic pathways have been shown to be essential, where knockouts of some genes involved in these processes result in severe growth defects, especially under nutrient limiting conditions [10,11]. Autophagy also functions directly in immunity to limit infection [7,8,12]. It follows that targeting of autophagy and UPS pathways through ETP could be highly effective in perturbing host cell during infection, and indeed many examples of this has been described, as reviewed in Langin et al*.* [5].

## General autophagy

Macroautophagy (henceforth autophagy) is essential for cellular homeostasis through intracellular constituent recycling and mediates degradation of large protein complexes, insoluble protein aggregates and dysfunctional organelles [11]. The mechanism behind autophagy is generally conserved in eukaryotes and involves the coordinated action of around 40 autophagy-related (ATG) genes to direct formation of the autophagosome, a double-membraned vesicle that envelops cytosolic material such as proteins and organelles destined for degradation [11,13,14]. In brief, during plant autophagy, ATG1 and ATG13 take in signals from Target of Rapamycin (TOR) kinase which is influenced by both developmental and nutritional signals, although it is to note that TOR-independence exists for oxidative or ER stress-related autophagy [15]. During TOR-dependent autophagy, TOR blocks autophagy by phosphorylating ATG13, which prevents its association with ATG1. Upon nutrient deficiency or other TOR-inactivating conditions, ATG13 is rapidly dephosphorylated which results in ATG1 binding. Additional hypo-and hyperphosphorylation events allow the assembly of the ATG1, ATG13 and the accessory subunits ATG11 and ATG101. The active ATG1 kinase complex then promotes the ATG9-mediated delivery of lipids and membrane sources derived from the ER to the developing phagophore. Nucleation and expansion of the phagophore also involves the VPS34 lipid kinase, which generates PI3P on the phagophore membrane. In parallel, a ubiquitin-like conjugation system (ATG5/ATG12/ATG16) drives the conjugation of ATG8 to PE, which decorates the autophagosome membrane. The autophagosome is further sealed by recruitment of SH3 domain-containing protein 2 (SH3P2) that stimulates phagophore curvature. Mature autophagosomes are delivered to the lytic vacuole for degradation by vacuolar hydrolases.

Many steps in this pathway have been shown to be modulated during plant–pathogen interactions, with excellent reviews summarizing the known examples [5,7,12]. Effectors from plant pathogens were also shown to target different components of the host autophagic machinery, strengthening the idea that autophagy is a key cellular process in immunity [16]. Autophagy has been shown play both pro- and anti-pathogen roles [7,8,12]. Thus, there must be a high degree of regulation behind plant-pathogen interactions. In this review, we focus on protein degradation via selective autophagy, and its role in adding specificity to plant immunity.

## Selective autophagy

**Figure 1:**
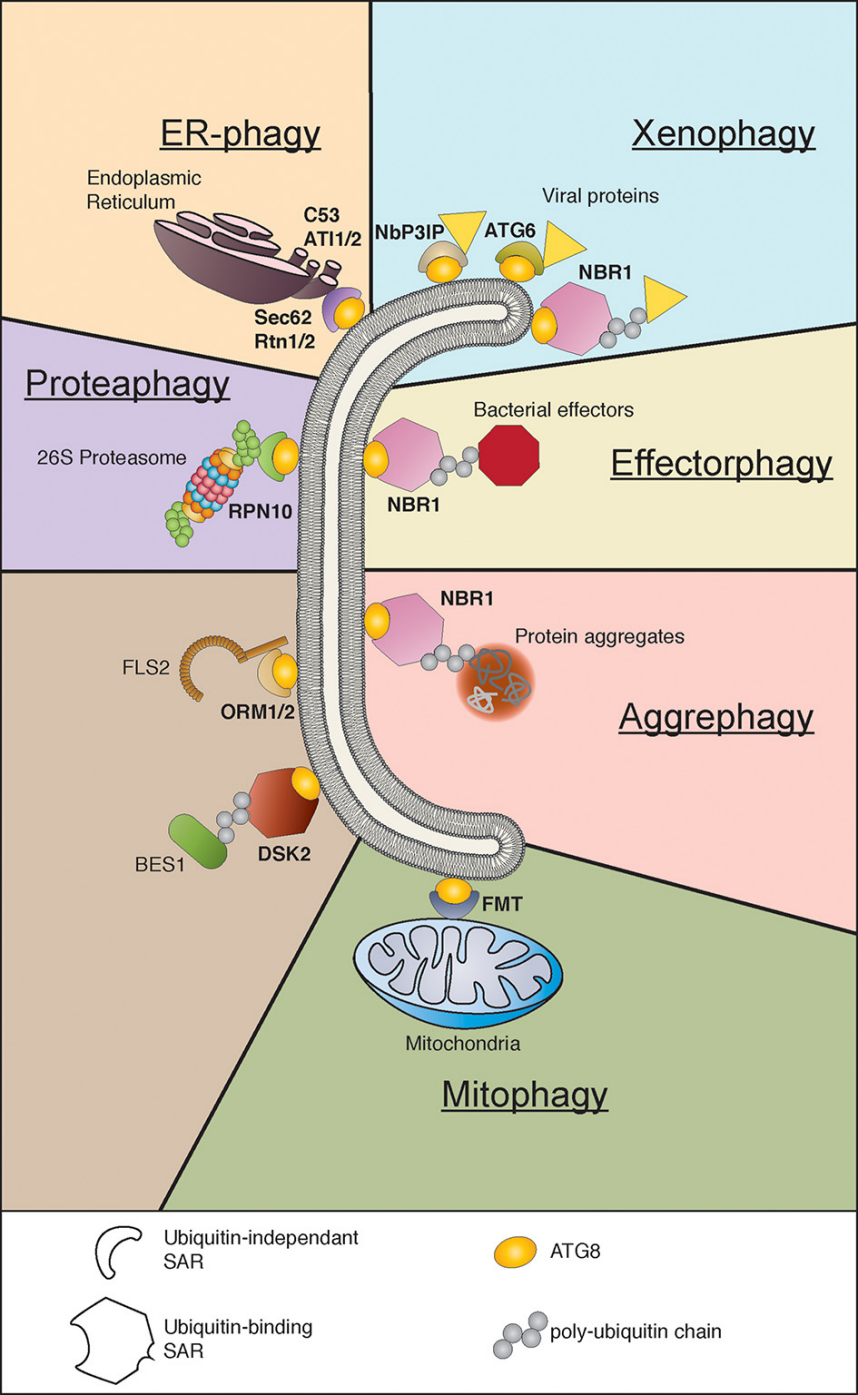
Selective autophagy is involved in the degradation of cellular and pathogenic components Selective autophagy receptors (SARs) bring their substrate to the nascent autophagosome ATG8 interaction. Substrate binding can be ubiquitin-dependent or -independent. The cargo for xenophagy and effectorphagy includes viral proteins and bacterial effectors. Protein aggregates are targeted in aggrephagy. Organelles such as endoplasmic reticulum (ER), proteasome, and mitochondria are the cargo for ER-phagy, proteaphagy and mitophagy respectively. Specific proteins such as FLS2 and BES1 have also been found to be targeted by SARs. Examples were highlighted based on data availability on ubiquitin-binding and AIM/LIR/UIM-containing. For a comprehensive review on receptors and their substrates see [19,20,25].

Our early understanding of autophagy characterized it as a non-specific “bulk” process, where cell cytoplasmic contents are “randomly” engulfed and degraded. Although it was already suggested then that autophagy displays selectivity toward cargo [17], only with the characterization of selective autophagy receptors (SARs, abbreviation not to be confused with systemic acquired resistance) there is strong proof of substrate specificity in autophagy [18]. Receptors have been identified for the selective autophagy of many cellular components across a range of eukaryotic organisms, for example proteasomes (proteaphagy), endoplasmic reticulum (ER-phagy), intracellular pathogens (xenophagy), protein aggregates (aggrephagy) and mitochondria (mitophagy) [19–22] (Figure 1). Particularly relevant to immunity, xenophagy of intracellular bacteria, viruses, protozoa and fungi have been well-described in mammalian systems, whereas for plants most research is directed at xenophagy of viruses [7,8,23,24]. Research on receptors and their identified substrates have been well-summarized for plants [20,25] and for the yeast model system [19]. Thus, in this review, we rather summarize common patterns behind domain organization of SARs and highlight recent advances in plant research regarding selective autophagy and immunity.

## The selective autophagy receptor

**Figure 2:**
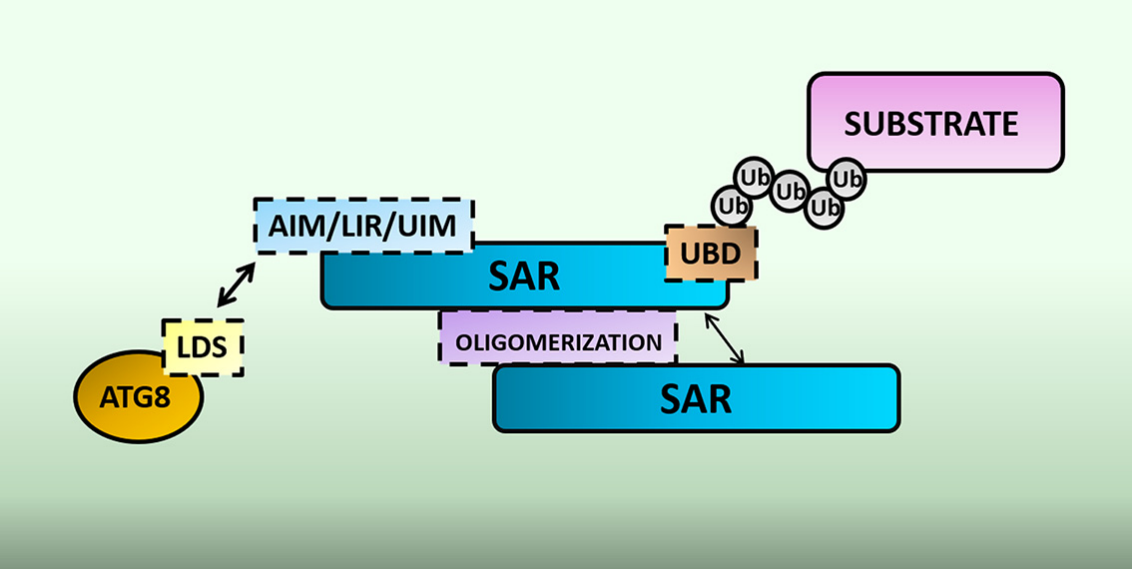
Domain organization of selective autophagy receptors (SAR) SARs can have ubiquitin-binding domains (UBD) that aids in substrate recognition. ATG8-, LC3- or ubiquitin- interacting motif (AIM/LIR/UIM) of the SAR contacts the LC3 docking site (LDS) on ATG8. Some SARs also contain domains that promote oligomerization.

One component underlying the mechanism of action behind selective autophagy is the receptor, and the study of these SARs has been a substantial driving force behind our understanding of selective autophagy. Despite the wide range of targets and functions, there are nonetheless common patterns that can be established in the mechanism of selective autophagy. In principle, SARs should be able to achieve two tasks. First—to recognize its substrate, either directly or via polyubiquitin chains on the substrate, and second—be able to bring the bound substrate to the autophagy machinery by binding ATG8, for example via ATG8-interacting motifs (AIMs), LC3-interacting motifs (LIR) or ubiquitin-interacting motifs (UIM) [19,20,25–27] (Figure 2).

### Ubiquitin-dependent substrate recognition

Many SARs recognize polyubiquitinated substrates, and contain ubiquitin-binding domains for the recognition of their target substrate [26]. The post-translational modification ubiquitination, also known as ubiquitylation, is described as “eat me” signal due to its role in targeting proteins toward degradative processes [28]. Ubiquitin is conjugated on a lysine residue of a target protein based on the enzymatic cascade of ubiquitin-activating (E1), ubiquitin-conjugating (E2) and ubiquitin-ligase (E3) enzymes [29]. The presence of internal lysine residues allows further conjugation of ubiquitin with itself to form polyubiquitinated chains which mark proteins for degradation [28], although targeting toward non-degradative processes such as the regulation of protein interactions and signal transduction also possible [29,30]. Domains known to bind ubiquitin include UBA (ubiquitin associated), UIM (ubiquitin-interacting motif), CUE (coupling of ubiquitin conjugation to endoplasmic reticulum degradation), TOM (target of Myb) and more as reviewed in [31].

One well-characterized plant SAR is NEIGHBOR OF BRCA1 gene 1 (NBR1), also known as Joka2 in solanaceous plants, which is a homolog of mammalian NBR1 and functionally similar to p62 [32,33]. NBR1 contains two UBA domains, but it was shown that only the C-terminal UBA domain of *A. thaliana* NBR1 could bind ubiquitin, and is thought to be involved in recognition of ubiquitinated targets for selective autophagy [32].

DOMINANT SUPPRESSOR OF KAR 2 (DSK2) is another SAR which acts as a ubiquitin receptor for BRI1-EMS SUPRESSOR 1 (BES1), the master regulator of the brassinosteroid (BR) pathway. Degradation of BES1 by DSK2 requires its UBA domain that can bind polyubiquitin chains, and AIM motif [34].

Similarly, proteasome subunit RPN10 which directs the 26S proteasome towards selective autophagy can bind both ubiquitin and ATG8 [35]. Additionally some proteasome subunits were found to be ubiquitinated thus strengthening RPN10 as a ubiquitin-dependent SAR [35,36].

Considering the role of ubiquitination as a degradation signal and the ubiquitin-binding ability of some SARs, many selective autophagy targets likely undergo ubiquitination. In fact, ubiquitinated proteins are observed to hyperaccumulate in *nbr1* or *atg5* mutants upon infection conditions shown to induce autophagy [37]. Thus, we hypothesize there are additional SARs with ubiquitin binding ability, and investigate this in more detail below.

### Ubiquitin-independent substrate recognition

In plant-pathogen interactions, there is evolutionary pressure on the host side to recognize pathogenic components, and on the pathogen side to escape this recognition. Indirect substrate recognition via ubiquitination is intuitively an effective strategy in which xenophagy-related SARs can participate in the xenophagy of a wide range of molecules, from viral capsids to oomycete and bacterial effectors. However, there are exceptions where substrate recognition is still achieved independently of ubiquitin association.

The turnip mosaic virus (TuMV) subunit P4 is recognized by the *A. thaliana* NBR1, and this interaction is still observed with a truncated NBR1 variant missing its UBA domain, suggesting for ubiquitin-independence in the interaction [38].

A study from our lab showed that a bacterial effector XopL is recognized by NBR1 through both ubiquitin-dependent and independent mechanisms. We show through co-immunoprecipitation that XopL interacted with full-length NBR1 and UBA deletion mutants, but a higher molecular weight species hypothesized to be ubiquitinated XopL, only interacted with NBR1 when a functional UBA domain is present [39].

Both these examples of ubiquitin-independence during NBR1-mediated xenophagy are intriguing as NBR1 is the only xenophagy receptor identified so far in plants. Are there more similar examples to be discovered, and if so how would NBR1 recognize the diverse effectors and viral subunits that could be present within the host cell? Are there some conserved features such as structure, that can drive the recognition of these foreign molecules by xenophagy receptors? Indeed, this could be a possibility, given that plant ER-phagy receptors ATG8-interacting proteins 1 and 2 (ATI1/2) share structural similarity with other ER-phagy receptors, where their AIM is located at the end of long, intrinsically disordered cytosolic regions [40]. The structure to function relationship is proposed as the disordered domain could bridge the membrane of the rough ER to ATG8 on phagophores or endolysosomes [40].

Although not shown to be a SAR, exocyst subunit Exo70B2 was found to interact with ATG8 via two AIMs, and is subject to autophagic transport to the vacuole upon treatment with defense-related compounds salicylic acid (SA) and benzothiadiazole (BTH) or immunogenic peptide flg22 [41]. We speculate that Exo70B2 could function as a SAR, directing cargo toward autophagic degradation. Interestingly, Exo70B2 is phosphorylated by the immunity-related kinase MPK3 that enhances its interaction with ATG8 [41]. Thus, if Exo70B2 acts as a SAR, this phosphorylation could present a novel way in which SARs are regulated.

Although not related to immunity, plant mitophagy receptor, FRIENDLY (FMT) does not have UBA domain yet mediates the recycling of damaged mitochondria in Arabidopsis [42]. Furthermore, plant ER-phagy receptors ATI1/2, Sec62, C53, reticulon proteins 1 and 2 (Rtn1/2) and degrade specific proteins in the ER during proteotoxic stress [43–45] or dark-induced starvation [46]. These proteins do not have any predicted ubiquitin-binding functions, thus future work should confirm their ubiquitin-independence and characterize the mechanism.

For mammalian systems as well, under specific conditions receptors link their cargo to autophagosomes during mitophagy, pexophagy, and virophagy in a ubiquitin-independent manner [47]. Thus, is entirely possible that there are more examples of ubiquitin-independent selective autophagy involved in immunity.

### Motifs for ATG8 interaction

Once a target substrate is bound, the subsequent role of a SAR is to bring its bound substrate to the autophagic machinery, usually the nascent autophagosome. To achieve this, canonical ATG8-interacting motif (AIM), otherwise known as LC3-interacting region (LIR), or the non-canonical ubiquitin-interacting motif (UIM) on a SAR is crucial, since the autophagosome is decorated with ATG8 proteins to facilitate this specific binding [48,49]. Mechanistically, this binding between the SAR and ATG8 occurs as the AIM/LIR contacts a hydrophobic patch on ATG8 known as the LIR docking site (LDS) [50,51].

Plant NBR1 has a canonical AIM facilitating its interaction with ATG8 on autophagosomes [32,33]. Svenning et al. additionally showed that AtNBR1 during co-expression with AtATG8 or human GABARAPL2, required its AIM to be recognized as an autophagic substrate in HeLa cells [32].

Many other SARs have been found to contain AIMs which are crucial for their function in selective autophagy, for example as mentioned above DSK2, RPN10 and potentially Exo70B2 [34,35,41].

RPN10 was additionally shown to have a UIM, which is a non-canonical motif for ATG8 interaction named for its similarity to existing UIM [35,52]. The authors further screened known ATG8-binding proteins for UIM, and identified three PUX proteins, thus raising the possibility that novel SARs with UIM are yet to be characterized [52].

The importance of the AIM in autophagy has inspired tools for AIM prediction to aid in discovery of novel autophagy targets [53,54]. This has potential in aiding in the identification of novel SARs, and will be discussed below.

### Oligomerization

Oligomerization of SARs have been shown to be especially important during selective autophagy of aggregates [55,56]. This is because protein aggregates are present as biomolecular condensates, which are membraneless assemblies where certain cellular components such as biopolymers are concentrated [57]. Domains such as Phox and Bem1p (PB1) are present on some SARs which enable their oligomerization [58]. It is rather intuitive that SARs involved in aggrephagy would oligomerize, so that they more easily localize into condensates where their targets reside. Indeed, this idea holds true when we consider the receptors Dsk2 and Cue2, which play a role in both proteasome- and autophagy-dependent protein degradation [59]. Lu and colleagues designed artificial ubiquitin- and Atg8-binding receptors, with or without the ability to oligomerize, and found that only oligomeric receptors could function in aggrephagy. Although direct evidence was not shown, a previous study suggest that Joka2 aggregates contain its multimeric form, and that in the nucleus and cytoplasm it is in a monomeric form [60].

The ubiquitin-binding domains of SARs can also contribute to their oligomerization. For example, a PB1 domain is present on plant NBR1, which allows it to form oligomers with other molecules of itself, but this oligomerization is also partially dependent on its UBA domains [32,60]. In a similar way, human p62 is the main driver of ubiquitin condensate formation, but NBR1 also promotes the p62 condensation through its PB1 and UBA domains [61].

SAR oligomerization may have wider implications beyond aggrephagy, and may function generally in promoting the interaction with ATG8. It was shown that oligomerization of mammalian p62 has an important role in stabilizing the binding to LC3 on the autophagosome membrane [55,56]. Furthermore, the DIX domain of Dishevelled (Dvl) mediates its self-oligomerization, which is ultimately required for interaction with LC3 [62]. There is also increasing evidence that autophagosome formation during autophagy involves liquid-liquid phase separation, which can be promoted by the oligomerization of SARs [63].

## Recent advances in selective autophagy in the context of plant immunity

### Plant–viral interactions

The pro-plant role of xenophagy during plant–viral interactions is well-characterized. The SAR NBR1 functions in the targeted degradation of viral particles and proteins. For example, cauliflower mosaic virus (CaMV) capsid and P4 proteins, and Turnip mosaic virus (TuMV) HcPro have been shown to be removed by NBR1 [37,38]. In turn, TuMV evolved viral proteins VPg and 6K2 to counteract NBR1-driven host xenophagy by blocking the degradation of HcPro [64]. More recently, it has been reported that nuclear autophagy degrades geminivirus nuclear protein C1 to restrict viral infection in solanaceous plants [65]. In the present study, viral protein C1 harbors an AIM which facilitates its interaction with ATG8, leading to its degradation via autophagy in the cytoplasm. Considering that AIM-containing proteins can act as autophagy adaptors or receptors it is tempting to speculate whether C1, similar to *Phytophtora infestans* effector PexRD54 elaborated on below, functions to target nuclear proteins upon viral infection.

A recent study identified that autophagy core component ATG6/Beclin1 might act as a SAR, as it mediates the degradation of a TuMV RNA-dependent RNA polymerase Nlb to restrict virus infection [66]. ATG6/Beclin1 was also shown to be involved in autophagy-like protein degradation of tobacco calmodulin-like protein rgs-CaM which bound to viral RNA silencing suppressor proteins, thus enhancing host antiviral RNAi [67]. Additionally, Jiang et al. identified a new cargo receptor NbP3IP with a previously unknown function, which specifically interacts with the P3 protein (VSR) of Rice stripe virus (RSV) and NbATG8f. These interactions mediate the selective degradation of the P3 protein and limit RSV infection [68].

Pro-viral effects of selective autophagy have also been described. P0 from Turnip yellows virus (TuYV) was found to manipulate selective autophagy for the degradation of ARGONAUTE1 (AGO1), exploiting ATI1 and ATI2 mediate the delivery of host AGO1 from the ER to the vacuole, ultimately down-regulating host antiviral RNA silencing [69].

Although the majority of the studies identified how SARs contribute to plant–virus interactions, further mechanistic details, such as oligomerization state of the SAR or whether substrate recognition is mediated by ubiquitin, remain still elusive.

### Plant–bacterial interactions

During interactions with bacteria, plant autophagy can also function in a pro-plant or pro-bacterial manner. FLAGELLIN-SENSING 2 (FLS2), a receptor kinase which recognizes bacterial flagellin, was found to be degraded by selective autophagy [70]. Orosomucoid (ORM) proteins ORM1 and 2 act as SARs in the degradation of non-activated FLS2, which ensures sufficient amounts of activated FLS2 for signaling [70]. The pathway is hijacked for pro-pathogen effects, for example during *Pseudomonas syringae* infection, where it was found that a T3E HopM1 causes the targeted degradation of the host proteasome by inducing proteaphagy, which could involve the proteaphagy receptor RPN10 since *Pst* bacterial growth was elevated in the *rpn10* mutant [37].

As viruses are the only described intracellular pathogens in plants, we propose that xenophagy includes degradation of viruses, “virophagy” and of effectors, “effectorphagy”. During effectorphagy, bacterial effectors are recognized in the plant cell and degraded via the autophagy pathway, for example as in Leong et al. [39]. The study showed that NBR1/Joka2 interacts with and causes the autophagic degradation of a *Xanthomonas campestris* pv. *vesicatoria (Xcv)* effector XopL which are present in aggregates, providing a link between aggrephagy in immunity [39]. This is a novel example in plants of selective autophagy targeting bacterial effectors, and there is a possibility that NBR1 is involved in degradation of other bacterial effectors. Evidence for this include increased bacterial growth and disease progression of *Pseudomonas syringae* pathovar *tomato* DC3000 *(Pst)* in the *nbr1* mutant compared with WT, which indicates for a role of NBR1 in *Pst* infection [37]. The same study further shows that overexpression of NBR1 reduces water soaking caused by HopM1. Both of these results led the authors to speculate that NBR1 is involved in the degradation of other *Pst* effectors.

### Plant–oomycete interactions

*Phytophtora infestans*, the causal pathogen of potato late blight, was shown to secrete an effector PexRD54 which, via its AIM motif, associates with ATG8CL to antagonize Joka2, ultimately enhancing oomycete virulence [71]. Further studies showed that this interaction between oomycete effector and host SAR takes place at the extrahaustorial membrane (EHM), where defense-related Rab8a-vesicles labelled with Joka2 and ATG8CL accumulated [72,73]. Apart from providing more insight to the role of autophagy during plant–oomycete interactions, the authors additionally developed a tool for autophagy suppression. The AIM from PexRD54 was used to create an AIM peptide, which was shown to compete with ATG8-binding and thus blocks autophagy progression. The peptide has already been applied in competition assays to reinforce AIM-dependent ATG8 interactions identified in an immunoprecipitation coupled to mass spectrometry (IP-MS) screen [43]. It was also used to block autophagy by transient expression in *Nicotiana benthamiana*, as it is more specific to autophagy compared with other tools such as Concanamycin A which inhibits vacuolar degradation [39]. In summary, this highlights that studying the role of autophagy in the context of plant–microbe interaction may result in the discovery of novel tools to study the pathway in more detail.

## Novel perspectives on plant immunity introduced by selective autophagy research

### Identification of new receptors

We have established that SARs mediate substrate specificity during selective autophagy. Identifying and characterization of new SARs related to immunity can help advance our understanding of plant–pathogen interactions. We propose to identify new plant immunity-related SARs by mining the *Arabidopsis* database for proteins that bind to ubiquitin and/or ATG8, and further investigate these candidates by looking into their gene expression during infection using publicly available data.

Ubiquitin-binding proteins were identified based on the presence of characterized ubiquitin-binding domains (INTERPRO/PFAM/SMART) or with the Gene Ontology annotation “ubiquitin-binding” (GO:0043130). From these 106 ubiquitin-binding proteins, we further cross-checked previously published screens for ATG8-interacting proteins [52,53]. We identified 25 ubiquitin-binding proteins that was also found in Marshall et al.’s UIM-interaction dataset [52], and 15 that were found in Jacomin et al.'s LIR prediction for *A. thaliana* proteome [53], with 5 proteins found in both datasets (Table 1). Among the identified receptors include the known SARs NBR1/Joka2, RPN10 and DSK2 with both ubiquitin-binding and ATG8-interacting abilities (Table 1).

**Table 1:** Ubiquitin-binding proteins identified based on presence of ubiquitin-binding domains or Gene Ontology annotation

Entry	Entry.name	Protein.names	Gene. names	Length	Agi_ Code	UBQ. binding. Prediction	ATG8_ Y2H	iLIR. prediction
Q9SIV2	PSD2A_ARATH	26S proteasome non-ATPase regulatory subunit 2 homolog A (26S proteasome regulatory subunit RPN1a) (AtRPN1a) (26S proteasome regulatory subunit S2 homolog A)	RPN1A	891	AT2G20580	GO		
Q9ZT95	R7SL1_ARATH	Ubiquitin domain-containing protein 7SL RNA1 (At7SL-1)	7SL1	275	AT4G02970	GO		
Q9ZQZ6	R7SL2_ARATH	Ubiquitin domain-containing protein 7SL RNA2 (At7SL-2) (Protein ETERNALLY VEGETATIVE PHASE 1)	EVE1	263	AT4G03350	GO		
Q9ZQZ4	Q9ZQZ4_ARATH	Putative ubiquitin-like protein (Ubiquitin family protein) (Uncharacterized protein AT4g03370)	At4g03370	295	AT4G03370	GO		+
Q9XIP2	OTU6_ARATH	OVARIAN TUMOR DOMAIN-containing deubiquitinating enzyme 6 (OTU domain-containing protein 6) (EC 3.4.19.12) (Deubiquitinating enzyme OTU6) (Otubain-like deubiquitinase 1)	OTU6	505	AT2G27350	UBA		
Q9SZD6	PETS_ARATH	Polyprotein of EF-Ts, chloroplastic (150 kDa pro-protein) (Protein EMBRYO DEFECTIVE 2726) [Cleaved into: Plastid-specific ribosomal protein-7, chloroplastic; Elongation factor Ts, chloroplastic (EF-Ts)]	PETs	953	AT4G29060	UBA-like		
Q9SUG6	PUX3_ARATH	Plant UBX domain-containing protein 3 (PUX3) (CDC48-interacting UBX-domain protein 3)	PUX3	302	AT4G22150	UBX	+	
Q9SSD3	Q9SSD3_ARATH	F18B13.13 protein (Ubiquitin system component Cue) (Uncharacterized protein At1g80040)	At1g80040	248	AT1G80040	CUE		+
Q9SJJ5	CKS2_ARATH	Cyclin-dependent kinases regulatory subunit 2	CKS2	83	AT2G27970	GO		
Q9SB64	NBR1_ARATH	Protein NBR1 homolog (AtNBR1) (At4g24690)	NBR1	704	AT4G24690	UBA	+	
Q9M9U7	Q9M9U7_ARATH	At1g18760 (F6A14.13 protein) (Zinc finger, C3HC4 type (RING finger) family protein)	At1g18760	224	AT1G18760	UIM		
Q9M548	DRM2_ARATH	DNA (cytosine-5)-methyltransferase DRM2 (EC 2.1.1.37) (Protein DOMAINS REARRANGED METHYLASE 2)	DRM2	626	AT5G14620	UBA		
Q9M0X2	Q9M0X2_ARATH	Ubiquitin-like superfamily protein	At4g05230	206	AT4G05230	GO		
Q9M0X1	Q9M0X1_ARATH	Ubiquitin-like superfamily protein	At4g05240	197	AT4G05240	GO		
Q9M0X0	Q9M0X0_ARATH	Ubiquitin-like superfamily protein	At4g05250	318	AT4G05250	GO		
Q9M0W9	Q9M0W9_ARATH	Ubiquitin-like superfamily protein (Uncharacterized protein AT4g05260)	At4g05260	259	AT4G05260	GO		
Q9M0W8	Q9M0W8_ARATH	Ubiquitin-like superfamily protein (Uncharacterized protein AT4g05270)	At4g05270	129	AT4G05270	GO		
Q9M0W4	Q9M0W4_ARATH	Ubiquitin-like superfamily protein	At4g05310	415	AT4G05310	GO		
Q9M0N1	PUX10_ARATH	Plant UBX domain-containing protein 10 (PUX10)	PUX10	480	AT4G10790	UBA/UBX	+	
Q9LYE5	CID5_ARATH	Polyadenylate-binding protein-interacting protein 5 (PABP-interacting protein 5) (Poly(A)-binding protein-interacting protein 5) (PAM2-containing protein CID5) (Protein CTC-INTERACTING DOMAIN 5) (Protein INCREASED POLYPLOIDY LEVEL IN DARKNESS 1)	CID5	155	AT5G11440	CUE		
Q9LYC2	NPL41_ARATH	NPL4-like protein 1	At3g63000	413	AT3G63000	NPL4		
Q9LXE5	DRM1L_ARATH	DNA (cytosine-5)-methyltransferase DRM1 (EC 2.1.1.37) (Protein DOMAINS REARRANGED METHYLASE 1)	DRM1	624	AT5G15380	UBA		
Q9LVR6	DAR3_ARATH	Protein DA1-related 3	DAR3	450	AT5G66640	DA1-like		
Q9LPL6	TOL3_ARATH	TOM1-like protein 3	TOL3	506	AT1G21380	VHS-GAT		+
Q9LNC6	TOL2_ARATH	TOM1-like protein 2	TOL2	383	AT1G06210	VHS-GAT		+
Q9LJN8	BUB31_ARATH	Mitotic checkpoint protein BUB3.1 (Protein BUDDING UNINHIBITED BY BENZYMIDAZOL 3.1)	BUB3.1	340	AT3G19590	WD40		
Q9LHS5	Q9LHS5_ARATH	Cell division related protein-like (DnaJ and myb-like DNA-binding domain-containing protein)	At5g06110	663	AT5G06110	UBD		
Q9LHN3	Q9LHN3_ARATH	AT3g18860/MCB22_3 (Transducin family protein / WD-40 repeat family protein)		760	AT3G18860	WD40/PFU/ PUL	+	+
Q9LHG8	ELC_ARATH	Protein ELC (AtELC) (ESCRT-I complex subunit VPS23 homolog 1) (Protein VACUOLAR PROTEIN SORTING 23A) (Vacuolar protein-sorting-associated protein 23 homolog 1)	ELC	398	AT3G12400	UEV		
Q9LFL3	TOL1_ARATH	TOM1-like protein 1	TOL1	407	AT5G16880	VHS-GAT		
Q9LET3	RBL20_ARATH	Rhomboid-like protein 20 (AtRBL20)	RBL20	293	AT3G56740	UBA		
Q9FMQ0	Q9FMQ0_ARATH	AT5g12120/MXC9_8 (At5g12120) (Ubiquitin-associated/translation elongation factor EF1B protein) (Uncharacterized protein At5g12120)	MXC9.8	619	AT5G12120	UBA		+
Q9FLZ3	KIN12_ARATH	SNF1-related protein kinase catalytic subunit alpha KIN12 (AKIN12) (EC 2.7.11.1) (AKIN alpha-3) (AKINalpha3) (SNF1-related kinase 1.3) (SnRK1.3)	KIN12	494	AT5G39440	UBA		
Q9FKZ1	DRL42_ARATH	Probable disease resistance protein At5g66900	At5g66900	809	AT5G66900	GO		
Q9FKZ0	DRL43_ARATH	Probable disease resistance protein At5g66910	At5g66910	815	AT5G66910	GO		
Q9FKN7	DAR4_ARATH	Protein DA1-related 4 (Protein CHILLING SENSITIVE 3)	DAR4	1613	AT5G17890	DA1-like		
Q9FJX9	DAR7_ARATH	Protein DA1-related 7	DAR7	560	AT5G66610	UIM	+	
Q9FJX8	DAR6_ARATH	Protein DA1-related 6	DAR6	644	AT5G66620	UIM	+	
Q9FFQ0	TOL5_ARATH	TOM1-like protein 5	TOL5	447	AT5G63640	VHS-GAT		
Q9FF81	VPS36_ARATH	Vacuolar protein sorting-associated protein 36 (AtVPS36) (ESCRT-II complex subunit VPS36)	VPS36	440	AT5G04920	VPS36		
Q9FDZ8	Q9FDZ8_ARATH	At1g73440 (Calmodulin-like protein) (Uncharacterized protein T9L24.51)	T9L24.51	254	AT1G73440	UIM	+	
Q9C9Y1	TOL8_ARATH	TOM1-like protein 8	TOL8	607	AT3G08790	VHS		
Q9C9U5	SIS8_ARATH	Probable serine/threonine-protein kinase SIS8 (EC 2.7.11.1) (MAPKK kinase SIS8) (Protein SUGAR INSENSITIVE 8)	SIS8	1030	AT1G73660	UIM	+	
Q9C701	BUB32_ARATH	Mitotic checkpoint protein BUB3.2 (Protein BUDDING UNINHIBITED BY BENZYMIDAZOL 3.2)	BUB3.2	339	AT1G49910	WD40		
Q9C5H4	MTV1_ARATH	Protein MODIFIED TRANSPORT TO THE VACUOLE 1	MTV1	690	AT3G16270	VHS-GAT		
Q9C5G7	PUX13_ARATH	Plant UBX domain-containing protein 13 (PUX13)	PUX13	525	AT4G23040	UBA/UBX	+	
Q9ASS2	FREE1_ARATH	Protein FREE1 (FYVE domain protein required for endosomal sorting 1) (FYVE domain-containing protein 1)	FREE1	601	AT1G20110	Experimentally proven	+	
Q94JZ8	PUX7_ARATH	Plant UBX domain-containing protein 7 (PUX7)	PUX7	468	AT1G14570	UIM/UBA/UBX	+	
Q93ZS6	Q93ZS6_ARATH	AT3g05090/T12H1_5 (Transducin/WD40 repeat-like superfamily protein) (Uncharacterized protein At3g05090)	LRS1	753	AT3G05090	WD40		
Q8W4Q5	RIN3_ARATH	E3 ubiquitin protein ligase RIN3 (EC 2.3.2.27) (RING-type E3 ubiquitin transferase RIN3) (RPM1-interacting protein 3)	RIN3	577	AT5G51450	CUE		
Q8W4F0	DAR1_ARATH	Protein DA1-related 1	DAR1	553	AT4G36860	UIM/DA1	+	
Q8VZS6	GIP1_ARATH	GBF-interacting protein 1	GIP1	567	AT3G13222	UBA-like		
Q8VYC8	RIN2_ARATH	E3 ubiquitin protein ligase RIN2 (EC 2.3.2.27) (AMF receptor-like protein 1A) (RING-type E3 ubiquitin transferase RIN2) (RPM1-interacting protein 2)	RIN2	578	AT4G25230	CUE		
Q8VWF1	SH3P2_ARATH	SH3 domain-containing protein 2 (AtSH3P2)	SH3P2	368	AT4G34660	SH3	+	
Q8RWY8	Q8RWY8_ARATH	Nucleic acid binding protein (Uncharacterized protein At1g27750)	At1g27750	1075	AT1G27750	GO		+
Q8RWU7	PUX4_ARATH	Plant UBX domain-containing protein 4 (PUX4) (CDC48-interacting UBX-domain protein 4)	PUX4	303	AT4G04210	UBX	+	
Q8LG98	OTU1_ARATH	OVARIAN TUMOR DOMAIN-containing deubiquitinating enzyme 1 (OTU domain-containing protein 1) (EC 3.4.19.12) (Deubiquitinating enzyme OTU1)	OTU1	306	AT1G28120	GO		
Q8LG11	Q8LG11_ARATH	Proline-rich cell wall protein-like (Ubiquitin-associated/translation elongation factor EF1B protein)	At5g53330	221	AT5G53330	UBA		
Q8L860	TOL9_ARATH	TOM1-like protein 9	TOL9	675	AT4G32760	VHS-GAT		+
Q8L6Y1	UBP14_ARATH	Ubiquitin carboxyl-terminal hydrolase 14 (EC 3.4.19.12) (Deubiquitinating enzyme 14) (AtUBP14) (TITAN-6 protein) (Ubiquitin thioesterase 14) (Ubiquitin-specific-processing protease 14)	UBP14	797	AT3G20630	UBA		
Q8H0T4	UPL2_ARATH	E3 ubiquitin-protein ligase UPL2 (Ubiquitin-protein ligase 2) (EC 2.3.2.26) (HECT-type E3 ubiquitin transferase UPL2)	UPL2	3658	AT1G70320	UIM/UBA	+	+
Q8GY23	UPL1_ARATH	E3 ubiquitin-protein ligase UPL1 (Ubiquitin-protein ligase 1) (EC 2.3.2.26) (HECT-type E3 ubiquitin transferase UPL1)	UPL1	3681	AT1G55860	UIM/UBA	+	+
Q84WJ0	DAR5_ARATH	Protein DA1-related 5	DAR5	702	AT5G66630	DA1-like		
Q84L33	RD23B_ARATH	Ubiquitin receptor RAD23b (AtRAD23b) (Putative DNA repair protein RAD23-1) (RAD23-like protein 1) (AtRAD23-1)	RAD23B	371	AT1G79650	UBA		+
Q84L32	RD23A_ARATH	Probable ubiquitin receptor RAD23a (AtRAD23a) (Putative DNA repair protein RAD23-2) (RAD23-like protein 2) (AtRAD23-2)	RAD23A	368	AT1G16190	UBA		+
Q84L31	RD23C_ARATH	Ubiquitin receptor RAD23c (AtRAD23c) (Putative DNA repair protein RAD23-3) (RAD23-like protein 3) (AtRAD23-3)	RAD23C	419	AT3G02540	UBA		
Q84L30	RD23D_ARATH	Ubiquitin receptor RAD23d (AtRAD23d) (Putative DNA repair protein RAD23-4) (RAD23-like protein 4) (AtRAD23-4)	RAD23D	378	AT5G38470	UBA		
Q7Y175	PUX5_ARATH	Plant UBX domain-containing protein 5 (PUX5)	PUX5	421	AT4G15410	UBA/UBX	+	
Q6NQK0	TOL4_ARATH	TOM1-like protein 4	TOL4	446	AT1G76970	VHS-GAT		
Q6NQH9	CID6_ARATH	Polyadenylate-binding protein-interacting protein 6 (PABP-interacting protein 6) (Poly(A)-binding protein-interacting protein 6) (PAM2-containing protein CID6) (Protein CTC-INTERACTING DOMAIN 6)	CID6	175	AT5G25540	CUE		
Q5XF75	EFTS_ARATH	Elongation factor Ts, mitochondrial (EF-Ts) (EF-TsMt)	EFTS	395	AT4G11120	UBA-like		
Q4V3D3	PUX9_ARATH	Plant UBX domain-containing protein 9 (PUX9)	PUX9	469	AT4G00752	UIM/UBA-like/ UBX	+	
Q38997	KIN10_ARATH	SNF1-related protein kinase catalytic subunit alpha KIN10 (AKIN10) (EC 2.7.11.1) (AKIN alpha-2) (AKINalpha2) (SNF1-related kinase 1.1) (SnRK1.1)	KIN10	512	AT3G01090	UBA		
Q38942	RAE1_ARATH	Protein RAE1 (RNA export factor 1)	RAE1	349	AT1G80670	WD40		
Q0WSN2	DAR2_ARATH	Protein DA1-related 2 (Protein LATERAL ROOT DEVELOPMENT 3)	DAR2	528	AT2G39830	UIM	+	
Q0WL28	Q0WL28_ARATH	Ubiquitin system component Cue protein (Uncharacterized protein At1g27750)	At1g27752	656	AT1G27752	CUE		+
P92958	KIN11_ARATH	SNF1-related protein kinase catalytic subunit alpha KIN11 (AKIN11) (EC 2.7.11.1) (AKIN alpha-1) (AKINalpha1) (SNF1-related kinase 1.2) (SnRK1.2)	KIN11	512	AT3G29160	UBA		
P55034	PSMD4_ARATH	26S proteasome non-ATPase regulatory subunit 4 homolog (26S proteasome regulatory subunit RPN10) (AtRPN10) (26S proteasome regulatory subunit S5A homolog) (Multiubiquitin chain-binding protein 1) (AtMCB1)	RPN10	386	AT4G38630	UIM	+	
P0DKI4	PUX14_ARATH	Putative plant UBX domain-containing protein 14 (PUX14)	PUX14	417	AT4G14250	UBX	+	
P0C7Q8	DA1_ARATH	Protein DA1 (Protein SUPPRESSOR OF LARGE SEED AND ORGAN PHENOTYPES OF DA1-1 1)	DA1	532	AT1G19270	UIM	+	
O82264	NPL42_ARATH	NPL4-like protein 2	At2g47970	413	AT2G47970	UBQ-like/MPN		
O81015	O81015_ARATH	At2g26920 (Ubiquitin-associated/translation elongation factor EF1B protein) (Uncharacterized protein At2g26920)	At2g26920	646	AT2G26920	UBA		+
O80996	BRIZ2_ARATH	BRAP2 RING ZnF UBP domain-containing protein 2 (EC 2.3.2.27)	BRIZ2	479	AT2G26000	Znf_UBP-like		
O80910	TOL6_ARATH	TOM1-like protein 6	TOL6	671	AT2G38410	VHS-GAT		
O65506	O65506_ARATH	Disease resistance protein (TIR-NBS-LRR class) (Putative disease resistance protein)	At4g36140	1607	AT4G36140	GO		
O48726	RPN13_ARATH	26S proteasome regulatory subunit RPN13 (AtRPN13) (26S proteasome non-ATPase regulatory subunit 13)	RPN13	300	AT2G26590	PH		
O48696	O48696_ARATH	AAA-type ATPase family protein (F3I6.23 protein)	At1g24290	525	AT1G24290	UBA		
O23249	CKS1_ARATH	Cyclin-dependent kinases regulatory subunit 1 (CKS1-At)	CKS1	87	AT2G27960	GO		
F4KED5	F4KED5_ARATH	Ubiquitin system component Cue protein	At5g32440	265	AT5G32440	CUE		
F4KCN6	F4KCN6_ARATH	Ubiquitin receptor RAD23 (DNA repair protein RAD23)	At5g16090	171	AT5G16090	UBA		
F4KAU9	TOL7_ARATH	TOM1-like protein 7	TOL7	542	AT5G01760	VHS-GAT		
F4JPR7	PUX8_ARATH	Plant UBX domain-containing protein 8 (PUX8) (Ara4-interacting protein) (Suppressor of ARA4-induced defect of ypt1) (SAY1)	PUX8	564	AT4G11740	UIM/UBA-like/UBX	+	+
F4JI85	F4JI85_ARATH	Ubiquitin family protein	At4g03360	322	AT4G03360	GO		
F4IXN6	PUX6_ARATH	Plant UBX domain-containing protein 6 (PUX6)	PUX6	435	AT3G21660	UBX	+	+
F4ITP6	F4ITP6_ARATH	Ubiquitin-like superfamily protein	At2g32350	242	AT2G32350	GO		
F4I241	BUB33_ARATH	Mitotic checkpoint protein BUB3.3 (Protein BUDDING UNINHIBITED BY BENZYMIDAZOL 3.3)	BUB3.3	314	AT1G69400	WD40		
F4HXZ1	BRO1_ARATH	Vacuolar-sorting protein BRO1 (BRO domain-containing protein 1) (AtBRO1)	BRO1	846	AT1G15130	BRO1		
A4FVR1	GIP1L_ARATH	GBF-interacting protein 1-like (Protein GIP1-like)	GIP1L	575	AT1G55820	UBA-like		
A0A1P8AW60	A0A1P8AW60_ARATH	Ubiquitin-associated (UBA)/TS-N domain-containing protein	At1g04850	324	AT1G04850	UBA		
Q9SII9	DSK2A_ARATH	Ubiquitin domain-containing protein DSK2a	DSK2A	538	AT2G17190	UBA	+	
Q9SII8	DSK2B_ARATH	Ubiquitin domain-containing protein DSK2b	DSK2B	551	AT2G17200	UBA		
Q8RXQ2	RBL18_ARATH	Rhomboid-like protein 18, AtRBL18	RBL18	287	AT2G41160	UBA		
Q9FT69	RQSIM_ARATH	ATP-dependent DNA helicase Q-like SIM, EC 3.6.4.12†(RecQ-like protein SIM, AtRecQsim, Similar to RecQ protein)	RECQSIM	858	AT5G27680	UBA		
Q500V3	Q500V3_ARATH	At2g12550 (Ubiquitin-associated (UBA)/TS-N domain-containing protein)	NUB1	562	AT2G12550	UBA		
Q8LB17	RBL15_ARATH	Rhomboid-like protein 15, AtRBL15, EC 3.4.21.-	RBL15	403	AT3G58460	UBA		
Q1EBV4	DDI1_ARATH	Protein DNA-DAMAGE INDUCIBLE 1, EC 3.4.23.-	DDI1	414	AT3G13235	UBA		

The list of proteins were cross-checked with previously published screens for ATG8-interacting proteins conducted by Marshall et al. [52] and Jacomin et al. [53] and indicated if they were found in these screens.

**Figure 3:**
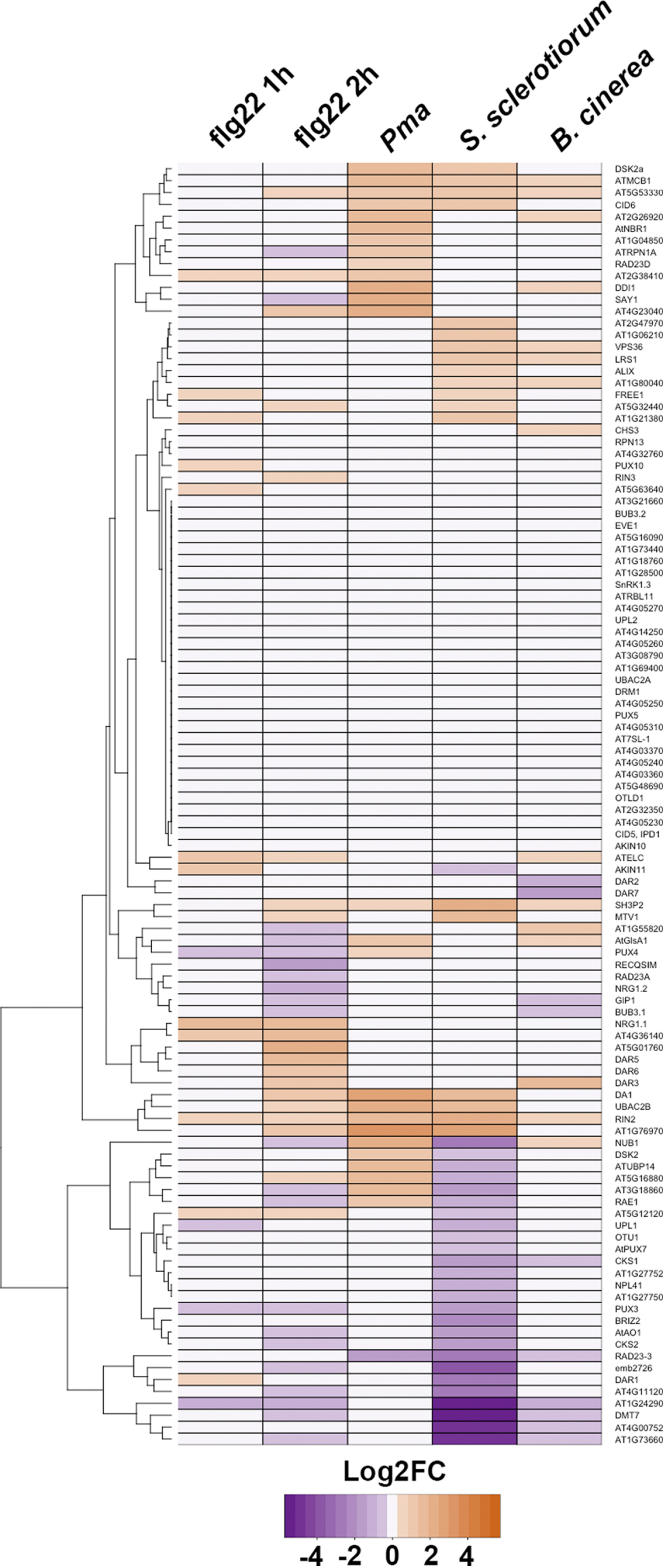
Differential gene expression of candidate SARs during immune responses represented in a heatmap Expression values are represented as Log2FC, and the color key is indicated at the bottom. The dendrogram on the left clusters together genes with similar expression pattern. Columns correspond to the different dataset extracted from the public online database (http://ipf.sustech.edu.cn/pub/athrna/) as following: flg22 1h (PRJNA491484); flg22 2h (PRJNA429781); *P. syringae* pv. *maculicola* (Pma) (PRJNA390966); *S. sclerotiorum* (PRJNA418121) and *B. cinerea* (PRJNA276444).

Expression level of candidate receptors were then analyzed using data available from public *Arabidopsis* RNAseq libraries [74]. In brief, five representative datasets were used for the analysis based on following criteria: (1) Dataset contains only one treatment. (2) There are at least three biological replicates in control and treated conditions. (3) Datasets are non-redundant. FPKM values for each gene and replicate were extracted from the five datasets, and subsequent Log2 Fold-change (Log2FC) and false discovery rate (FDR) for each gene comparing treated samples to control samples were assessed using Rstudio software (https://www.rstudio.com/). Only, genes with FDR < 0.05 were considered statistically significant. Log2FC of statistically significant genes are displayed as a heatmap with a dendrogram representing genes sorting based on expression pattern similarity from the different dataset (Figure 3).

As NBR1 is well-characterized, we first investigated the data on NBR1 presented by the heatmap. Notably, expression of NBR1 displayed a high positive log fold change during infection with P. *syringae* pv. *maculicola (Pma)* but showed no significant expression differences during flg22 treatment or *Sclerotinia sclerotiorum* and *Botrytis cinerea* infection (Figure 3).

We draw focus to the proteins which display expression patterns evidently different from NBR1, as they could be novel immune-related SARs with specialized functions different from the well-characterized NBR1. For example, the subset of proteins ATMCB1, VPS36, LRS1, SH3P2 and RIN2 were up-regulated during infection with *S. sclerotiorum* and *B. cinerea*, and could be novel ubiquitin- and/or AIM- binding receptors with functions in immunity. In contrast, there is a high negative log fold change in the expression of proteins AT1G24290, DMT7, ATG4G00752, and AT1G73660 during *S. sclerotiorum* and *B. cinerea* infection (Figure 3). This indicates for a possibility that these proteins could be targeted during infection, perhaps because they as well play a role in immunity.

*S. sclerotiorum* and *B. cinerea* are necrotrophic in contrast with the hemibiotrophic *P. syringae*. Past studies have shown autophagy plays different roles during immunity against biotrophic and necrotrophic pathogens, due in part to its cross-talk with salicylic-acid signalling which mediates resistance against biotrophic pathogens [75]. It is therefore plausible that these putative SARs which respond specifically to necrotrophic pathogens could mediate resistance in a novel manner.

We have identified here many ubiquitin- and/or ATG8- binding receptors with significant expression differences upon flg22 treatment or pathogen challenge. Further investigation should be directed at validating the role of these proteins as SARs, and characterizing how they mediate pathogen interactions and immune responses. The molecular mechanisms that drive their substrate recognition and interaction with autophagic machinery should also be studied so we gain more insight into these interactions.

### ATG8 isoforms

We have discussed in depth how SARs can promote the specificity of substrate degradation via autophagy. On the other hand, recent research has shown that different ATG8 isoforms can also add specificity to autophagy. This was already hinted at in earlier publications where Arabidopsis NBR1 showed different levels of interaction with different members from the ATG8 family of proteins [32]. A study into potato ATG8 isoforms showed that the isoforms display differential gene expression under a range of conditions, and interact with distinct sets of proteins with varying degrees of overlap, which suggests some degree of functional specialization of the isoforms [76]. In addition, a yeast-two-hybrid screen revealed differential preference of *A. thaliana* ATG8 isoforms to associate with the UIM [52]. In an immunity context, Leong et al. found that in *N. benthamiana* transcript levels of *NbATG8-2* increased earlier than that of *NbATG8-1* during *Xcv* infection [39]. *P. infestans* effector PexRD54 also showed a preference for ATG8CL over ATG8IL [72].

**Figure 4:**
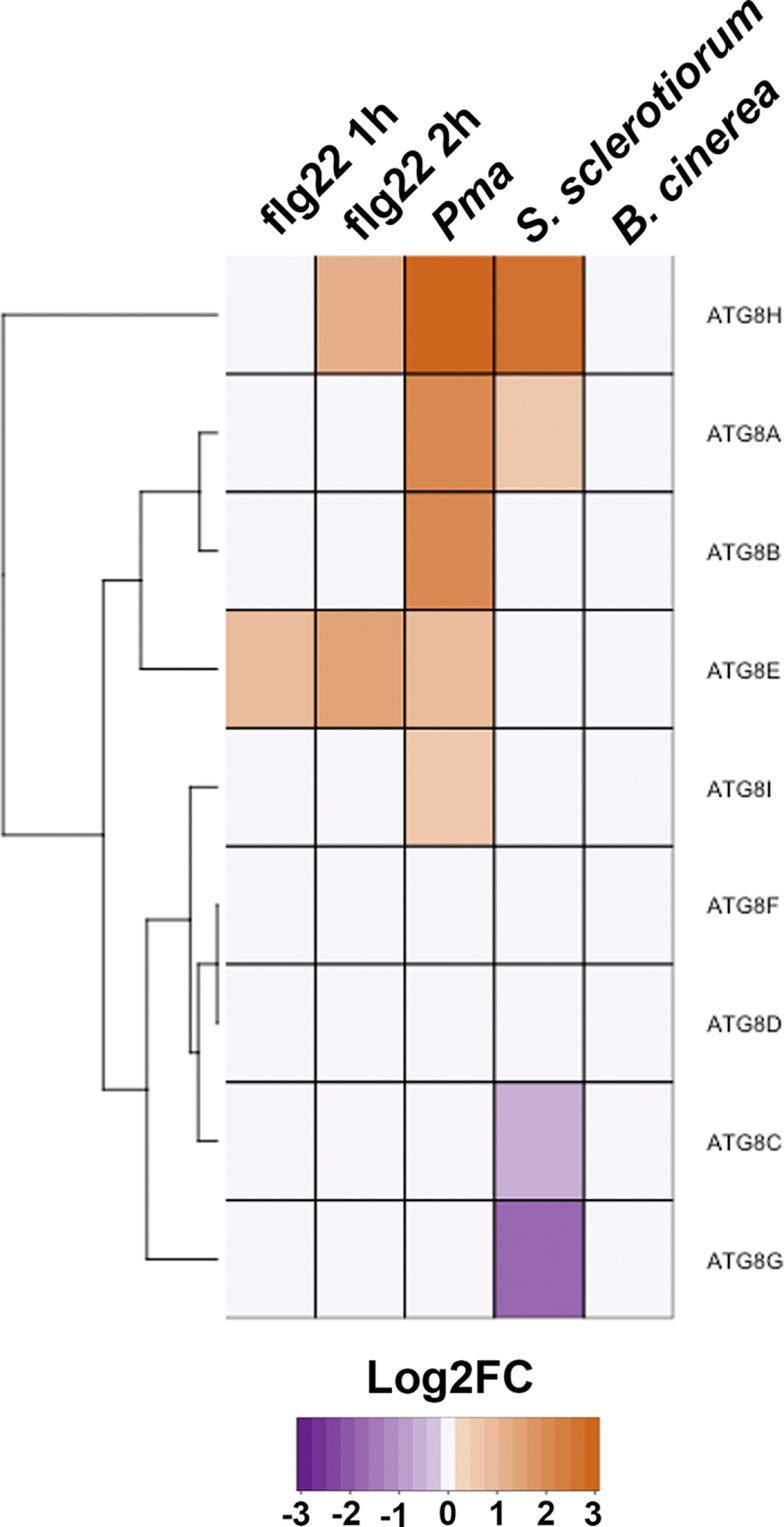
Differential gene expression of ATG8 isoforms during immune responses represented in a heatmap Expression values are represented as Log2FC, and the color key is indicated at the bottom. The dendrogram on the left clusters together isoforms with similar expression pattern. Columns correspond to the different dataset extracted from the public online database (http://ipf.sustech.edu.cn/pub/athrna/) as following: flg22 1h (PRJNA491484); flg22 2h (PRJNA429781); *P. syringae* pv. *maculicola* (Pma) (PRJNA390966); *S. sclerotiorum* (PRJNA418121) and *B. cinerea* (PRJNA276444).

To strengthen the idea that ATG8 isoforms add specificity during autophagic responses in immunity, we analyzed publicly available data on the gene expression of *A. thaliana* ATG8 isoforms during responses to immunogenic peptide flg22 and pathogens *Pma*, *B. cinerea*, and *S. sclerotiorum* (Figure 4). The analysis method and datasets used are the same as above for identification of putative novel SARs. We found differential gene expression patterns of the ATG8 isoforms during these conditions. Notably, the ATG8 isoforms show different expression patterns compared to each other and under different pathogenic contexts. As previously mentioned, potato ATG8 isoforms show differential gene expression and interact with distinct subsets of proteins, which suggests functionalization [76]. We build upon this hypothesis and apply it to our analysis of *A. thaliana* ATG8 isoforms, and suggest that the differential expression patterns in ATG8 isoforms strengthen the idea that the isoforms have specific functions. Some ATG8 isoforms could be grouped according to the similarity in which they behave. For example, *ATG8B, ATG8E*, and *ATG8I* were up-regulated during *Pma* infection and but did not show significant log fold change during *S. sclerotiorum* or *B. cinerea* infection. *ATG8A* and *ATG8H* responded to both hemibiotrophic *Pma* and necrotrophic *S. sclerotiorum*. In contrast, *ATG8C* and *ATG8G* displayed negative log fold change during infection with *S. sclerotiorum*. These patterns, where a subset of isoforms could specifically respond to the different pathogenic contexts, are similar to the patterns presented by putative ubiquitin- and/or ATG8- binding proteins analyzed above. This strongly supports a scenario where receptor and ATG8 works together to add specificity during immunity.

### Spatiotemporal control

Studies in selective autophagy and immunity have also revealed the importance of spatiotemporal regulation in cellular processes. Infection and autophagy are dynamic processes involving many players which constantly display changes in expression, localization, and stability. Dagdas et al. showed that during *P. infestans* infection, the effector PexRD54 caused a diversion of Joka2-ATG8CL defense-related vesicles to haustoria [72]. Leong et al. further showed that the protein accumulation of NBR1 occurs earlier than its increase in transcription [39]. In the same study, it was also shown that knockdown of NBR1 expression via virus-induced gene silencing (VIGS) reduced *Xcv* growth at 3 days-post-inoculation (dpi) compared with WT but not at 6dpi, thus indicating for a role in selective autophagy during the earlier steps of infection [39].

## Conclusions

Selective autophagy research has contributed much to our understanding of plant–pathogen interactions. During infection, autophagy is targeted by pathogens due to its central role in plant homeostasis but can also function in clearing pathogenic components and reducing infection. The SARs mediate selective autophagy, and studying these receptors gives us more mechanistic insight into how selective autophagy interacts with immunity. We have highlighted many excellent studies which have shed more light on the role of selective autophagy during immunity. There are nevertheless still many questions to answer, giving great potential for further investigation.

**Figure 5:**
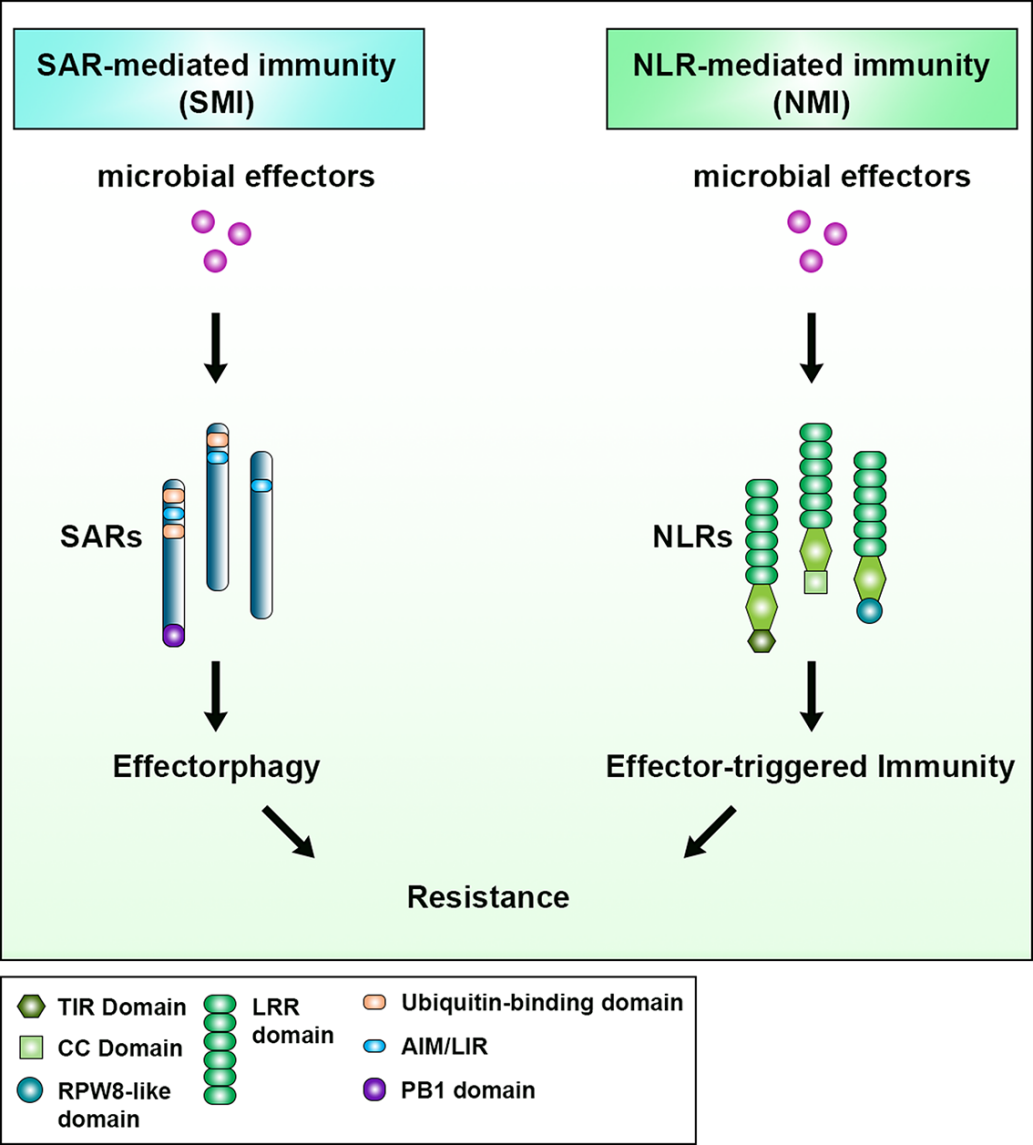
Parallels between SAR-mediated immunity (SMI) and NLR-mediated immunity (NMI) Both pathways depend on recognition of microbial effectors by receptors. SARs and NLRs are different by structure and domain organization. Both receptors inactivate the effector function either by inducing cell death (NMI) or by degrading the effector via autophagy (SMI) and leading to effector-triggered immunity.

In a classical immunity concept of the “zig-zag” model, gene-for-gene resistance is mediated by cell surface PRRs and cytoplasmic NLRs, which recognize pathogenic components in an extracellular and intracellular manner respectively [1,3]. In this review, we have highlighted the role of SARs in recognizing pathogenic components and their downstream effects within the cell. We draw parallels between NLRs-mediated immunity (NMI) and the role of immune-related SARs reviewed here, and propose that SARs can confer immunity to pathogens through SAR-mediated immunity (SMI) which results in effectorphagy (Figure 5). In both scenarios, effectors and/or their downstream effects are recognized by intracellular proteins with various features leading to effector-triggered immunity (ETI). In fact, Deretic et al. proposed, in the context of mammalian SARs, that they could represent a new paradigm in innate immunity as a new class of PRRs [77]. We support this view, and further emphasize the mechanistic details behind SAR function–how substrate recognition can be both ubiquitin-dependent and -independent, and how SARs directly bridge their substrate to the autophagosome thus immediately targeting them for degradation. This allows SARs to recognize a wide range of pathogenic effectors that are more directly involved in modulation of cellular processes.

## Summary

- Due to its central role in ensuring plant cellular homeostasis, autophagy is targeted by plant pathogens during infection
- Selective autophagy has been found to play pro-plant or pro-pathogen roles during immunity
- Similarities can be found across selective autophagy receptor domain organization and function
- Selective autophagy receptors are emerging as key players in plant interaction with bacteria, viruses, and oomycetes.
- The interplay between selective autophagy and pathogenic effectors can present new perspectives on the molecular mechanisms behind plant–pathogen interactions

## Abbreviations

CaMV: cauliflower mosaic virus
ETI: effector-triggered immunity
NMI: NLR-mediated immunity
PAMP: pathogen-associated-molecular-pattern
PRR: pattern-recognition receptor
PTI: PAMP-triggered immunity
SAR: selective autophagy receptor
SMI: SAR-mediated immunity
TuMV: Turnip mosaic virus
TuYV: Turnip yellows virus
VIGS: virus-induced gene silencing
